# Effects of *o*-vanillin on K^+^ transport of red blood cells from patients with sickle cell disease

**DOI:** 10.1016/j.bcmd.2014.02.004

**Published:** 2014-06

**Authors:** A. Hannemann, U.M.C. Cytlak, O.T. Gbotosho, D.C. Rees, S. Tewari, J.S. Gibson

**Affiliations:** a Department of Veterinary Medicine, University of Cambridge, Madingley Road, Cambridge, CB3 0ES, United Kingdom; b Department of Paediatric Haematology, King's College London School of Medicine, King's College Hospital NHS Foundation Trust, Denmark Hill, London, SE5 9RS, United Kingdom

**Keywords:** Sickle, Red blood cells, Aromatic aldehydes, *o*-Vanillin, Potassium permeability

## Abstract

Aromatic aldehydes like *o*-vanillin were designed to reduce the complications of sickle cell disease (SCD) by interaction with HbS, to reduce polymerisation and RBC sickling. Present results show that *o*-vanillin also directly affects RBC membrane permeability. Both the K^+^–Cl^−^ cotransporter (KCC) and the Ca^2 +^-activated K^+^ channel (or Gardos channel) were inhibited with IC_50_ of about 0.3 and 1 mM, respectively, with activities almost completely abolished by 5 mM. Similar effects were observed in RBCs treated with the thiol reacting reagent *N*-ethylmaleimide or with the Ca^2 +^ ionophore A23187, to circumvent any action via HbS polymerisation. The deoxygenation-induced cation conductance (sometimes termed P_sickle_) was partially inhibited, whilst deoxygenation-induced exposure of phosphatidylserine was completely abrogated. Na^+^/K^+^ pump activity was also reduced. Notwithstanding, *o*-vanillin stimulated K^+^ efflux through an unidentified pathway and resulted in reduction in cell volume (as measured by wet weight − dry weight). These actions are relevant to understanding how aromatic aldehydes may affect RBC membrane permeability *per se* as well as HbS polymerisation and thereby inform design of compounds most efficacious in ameliorating the complications of SCD.

## Introduction

The complications of sickle cell disease (SCD) are two-fold: a chronic anaemia subsequent to increased red blood cell (RBC) destruction and acute ischaemic signs following blockage of the microvasculature [1–3]. Signs depend on the organ involved and can be numerous. Severity, however, varies considerably between individuals. Notwithstanding this variability, all complications result from polymerisation of the abnormal form of haemoglobin, HbS, present in patients' RBCs. HbS has a single amino acid substitution at a critical site on the haemoglobin molecule [4,5]. At the β6 position, glutamic acid is replaced by valine and the loss of negative charge enables neighbouring HbS molecules to aggregate on deoxygenation, forming long rigid polymers which distort RBC shape and cause other deleterious abnormalities, including altered rheology, elevated membrane permeability and increased fragility [5].

At present, no specific treatment is available and management is usually supportive depending on whatever complication is most pronounced [1,3]. Recently, hydroxyurea has received attention as a drug of choice for ameliorating SCD complications [6–8]. Hydroxyurea's efficacy appears to depend on its ability to increase expression of fetal Hb, HbF—although other mechanisms may also be involved. HbF is not incorporated into HbS polymers and also serves to dilute the intracellular concentration of HbS, thereby reducing the tendency to polymerisation and sickling. Hydroxyurea is not without risks, however, being potentially teratogenic, with variable response, and also having issues of non-compliance [8]—factors which restrict its use to more severely affected individuals.

As a result, there is a continued search for other effective therapies. An alternative approach has been to reduce directly the tendency for HbS to polymerise on deoxygenation. In this context, a variety of aromatic aldehydes (and related compounds) have been tested, of which *o*-vanillin is a well known member [9–11]. These reagents form Schiff bases with HbS, increasing its oxygen affinity, and thereby reducing polymerisation and RBC sickling. Some have also been reported to interfere with RBC permeability [12], but although of potential importance, this aspect has not been widely investigated.

Several abnormal, or abnormally regulated, cation transporters participate in the pathogenesis of SCD [13–15]. These include the K^+^–Cl^−^ cotransporter (or KCC) and the Ca^2 +^-activated K^+^ channel (or Gardos channel), transport systems whose molecular identities are established. A third pathway, sometimes termed P_sickle_, is activated by HbS polymerisation and RBC shape change [13,16]. P_sickle_ is thought to function predominantly as a deoxygenation-induced cation pathway. Although it remains enigmatic at a molecular level, P_sickle_ will allow entry of Ca^2 +^ [17,18], and loss of Mg^2 +^ [19,20], with subsequent activation of the Gardos channel and perhaps KCC. The three pathways interact to mediate solute loss [15], thereby concentrating HbS, which greatly reduces the lag time for polymerisation upon deoxygenation—hence increasing the likelihood of sickling and ischaemia in the microvasculature.

In this report, radioactive tracer methodologies have been used to investigate the effects of *ortho (o)*-vanillin on K^+^ permeability, KCC, the Gardos channel and P_sickle_ in RBCs from SCD patients. Results show that this aromatic aldehyde markedly inhibits all three, as well as also affects HbS polymerisation and sickling, but also stimulates an unidentified K^+^ efflux pathway. These additional actions of *o*-vanillin may be of significant consideration when designing similar compounds to ameliorate the complications of SCD.

## Materials and methods

### Chemicals

Bumetanide, 3-[N-morpholino] propane sulfonic acid (MOPS), *N*-ethylmaleimide, ouabain, *ortho (o)*-vanillin, and salts were purchased from Sigma Chemical Co. (Poole, Dorset, UK). Clotrimazole and A23187 were purchased from Calbiochem (Nottingham, UK). ^86^Rb^+^ was supplied by Perkin Elmer (Beaconsfield, UK).

### Sample collection and handling

Blood samples were obtained by venepuncture of patients with sickle cell disease (SCD), both homozygous HbSS and heterozygous HbSC, with permission under ethical consent, using the anticoagulant EDTA. Samples were kept at 4 °C until use within 48 h.

### Solutions and tonometry

The standard saline (MBS) comprised (in mM): 145 NaCl, 1.1 CaCl_2_, 5 glucose and 10 MOPS, (pH 7.4 at 37 °C; 290 ± 5 mOsmol kg^ −1^ H_2_O). For experiments in which Cl^−^ dependence of K^+^ influx was examined, NO_3_^−^-containing salts replaced those containing Cl^−^. To prevent the rapid RBC shrinkage which would otherwise occur following maximal stimulation of the Gardos channel in experiments in which intracellular Ca^2 +^ was directly raised by incubation with the Ca^2 +^ ionophore A23187, a high-K^+^- and low-Ca^2 +^-containing saline was used, comprising (in mM): 80 KCl, 70 NaCl, 0.01 CaCl_2_, 0.15 MgCl_2_, 5 glucose and 10 MOPS. The wash solution to remove unincorporated ^86^Rb^+^ comprised isotonic MgCl_2_ (107 mM), buffered with MOPS (10 mM), pH 7.4 at 4 °C. Stock solutions of bumetanide (10 mM) were prepared in 100 mM Tris base and used at a final concentration of 10 μM. Stock solutions of ouabain (10 mM) were prepared in distilled water and used at a final concentration of 100 μM. Stocks of clotrimazole (CLT; 5 mM) were prepared in DMSO and used at final concentrations of 5 μM. After washing whole blood and removal of plasma and buffy coat, in most experiments RBC suspensions were placed in tonometers (Eschweiler, Kiel, Germany) at 20% haematocrit (Hct), to equilibrate at the requisite O_2_ tension. Tonometers were flushed with warm, humidified gas mixtures, supplied at the appropriate O_2_ tension using a Wösthoff gas mixing pump [21]. For CLT, dissolved in DMSO, appropriate controls were all treated with the same concentration of solvent (< 0.1% final).

### K^+^ flux measurements

To determine the activity of the K^+^ transport pathways, K^+^ influx was usually measured at 37 °C using ^86^Rb^+^ as a congener for K^+^ [22,23]. Cells were taken from the tonometers and diluted 10-fold into saline, pre-equilibrated at the appropriate O_2_ tension, at 260 mOsm kg^ −1^ and pH 7, conditions chosen in order to stimulate the K^+^–Cl^−^ cotransporter (KCC). ^86^Rb^+^ was added in 150 mM KNO_3_ to give a final [K^+^] of 7.5 mM in all experiments except those with HK saline and A23187-treated RBCs. After incubation with radioisotope for 10 min, RBCs were washed to remove extracellular ^86^Rb^+^, five-times in an ice-cold MgCl_2_ wash solution. For K^+^ efflux experiments, RBCs were loaded overnight at 4 °C by addition of ^86^Rb^+^ after which cells were washed five times in an ice-cold wash solution. RBCs were then suspended at 2% haematocrit (Hct) in standard saline at 37 °C. Aliquots were taken at 5 min intervals for 30–60 min and spun through phthalate oil. The cell pellet was lysed with detergent, deproteinised with TCA, and counted by liquid scintillation (cpm). A semilog plot (of cpm at time = t/cpm at time = 0) was used to determine the rate constant for K^+^ efflux. Except for experiments to measure Na^+^/K^+^ pump activity, ouabain (100 μM) and bumetanide (10 μM) were present in all experiments to obviate any K^+^ transport through the Na^+^/K^+^ pump and the Na^+^–K^+^–2Cl^−^ cotransporter, respectively. Either microhaematocrit determination or the cyanohaemoglobin method was used to measure the final Hct. KCC activity was assayed as Cl^−^-dependent K^+^ influx; Gardos channel activity as the CLT-sensitive (5 μM) K^+^ influx; Na^+^/K^+^ pump activity as the ouabain-sensitive (100 μM) K^+^ influx and P_sickle_ as the deoxygenation-induced K^+^ influx measured in the absence of Cl^−^.

### Labelling of phosphatidylserine exposure

For phosphatidylserine (PS) labelling, 5 μl aliquots (10^5^ RBCs) of each sample were placed in 250 μl of LA-FITC binding buffer and incubated in the dark at room temperature for 10 min. RBCs were then pelleted by centrifugation for 10 s at 16,100 *g*, washed once in LK or HK HBS to remove unbound LA-FITC and kept on ice until flow cytometry analysis. Unlike annexin-V, LA-FITC binds to PS in a Ca^2 +^-independent manner and control experiments showed that binding was irreversible. Inhibitors were tested for self-fluorescence at their highest concentration with unlabelled RBCs.

### FACS acquisition and analysis

Externalised PS was measured with FITC using an excitation wavelength of 488 nm in the FL1 channel with an emission wavelength of 519 nm on a fluorescence-activated flow cytometer (FACSCalibur, Becton Dickinson, BD) and analysed with the BD CellQuest Pro software using a protocol published previously [24]. Measurements were taken using a logarithmic gain. Forward scatter (FSC, size) and side scatter (SSC, granularity) gates for RBCs were identified in control experiments using anti-glycophorin A-PE labelled RBCs. The positive fluorescent gate was set using RBCs unlabelled with FITC-LA. For each measurement, 10,000 events were gated. PS positive cells were defined as all events falling within the preset FSC, SSC and positive fluorescent gates.

### Measurements of RBC sickling

RBCs were incubated in tonometers at 2% Hct for up to 60 min after which samples were fixed in the same solution as that used during incubation but with the addition of 0.3% glutaraldehyde. Control experiments showed that this protocol was sufficient to maintain the RBC shape for several weeks. Sickling was assessed by light microscopy. Several hundred RBCs (typically 300–400) were counted using an Improved Neubauer haemocytometer (in five 1 mm × 1 mm squares, the central one and the four corners).

### Measurement of cell water content

Cell water content was measured by the wet weight − dry weight method [25]. In brief, RBCs were pelletted by centrifugation at 12,000 *g* for 10 min at 4 °C. The extruded pellet was weighed immediately (to 0.01 mg) and again after drying for 18 h at 95 °C. Water content was expressed as ml water per g dry cell solids (ml/g dcs).

### Statistics

Results are presented as single observations representative of at least 3 others, or as means ± S.E.M. of *n* observations. Where appropriate, comparisons were made using paired Student's *t* tests, with *p* < 0.05 being considered significant.

## Results

### The effect of *o*-vanillin on sickling

**Fig. 1:**
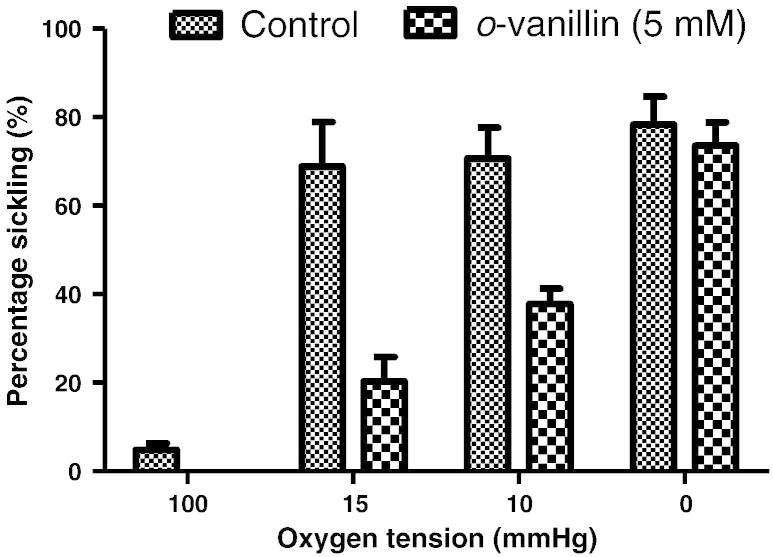
Sickling of red blood cells (RBCs) from homozygous (HbSS) patients with sickle cell disease (SCD). RBCs were incubated for 20 min in tonometers at 2% haematocrit at the oxygen tension indicated. Aliquots were then fixed in saline pre-equilibrated at the same oxygen tension with the addition of glutaraldehyde (0.3%). Morphological sickling was determined by light microscopy. Histograms represent means ± S.E.M., *n* = 3.

In the first series of experiments, the effect of *o-*vanillin (5 mM) was tested on sickling of RBCs from HbSS patients (Fig. 1). In fully deoxygenated RBCs, there was only a small reduction in percentage sickling (N.S.) in the presence of *o*-vanillin. At higher O_2_ tensions, nearer the P_50_ for O_2_ saturation of Hb, greater effects were observed, however, so that at an O_2_ tension of 15 mm Hg, sickling was inhibited by about 75% in the presence of *o*-vanillin (Fig. 1).

### The effect of *o*-vanillin on K^+^ transport in RBCs from SCD patients

**Fig. 2:**
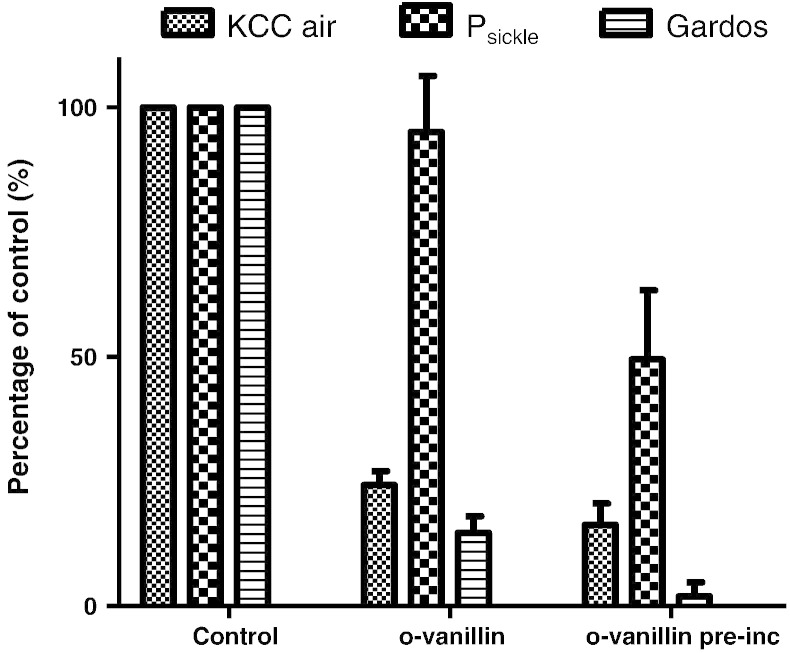
Effect of *o*-vanillin on the potassium permeability of RBCs from patients with SCD. Activity of the K^+^–Cl^−^ cotransport (KCC) was determined as the Cl^−^-dependent K^+^ influx in fully oxygenated RBCs from homozygous HbSS individuals. Activity of P_sickle_ was determined as the deoxygenation-induced Cl^−^-independent K^+^ influx in N_2_; that of the Gardos channel was determined as the clotrimazole (CLT; 5 μM)-sensitive K^+^ influx—both in fully deoxygenated RBCs. *o*-Vanillin (5 mM) was present either during the period of measurement of transporter activity only, or, in addition, during 30 min pre-incubation as well. Flux measurements are normalised to those measured in control RBCs in the absence of *o*-vanillin and presented as a percentage of their maximal value, which, for HbSS were 2.35 ± 0.45 mmol (l cells h)^−1^ for KCC in air, 1.02 ± 0.22 for P_sickle_ and 5.80 ± 1.16 for the Gardos channel. Data represent means ± S.E.M. for 4–7 determinations.

**Fig. 3:**
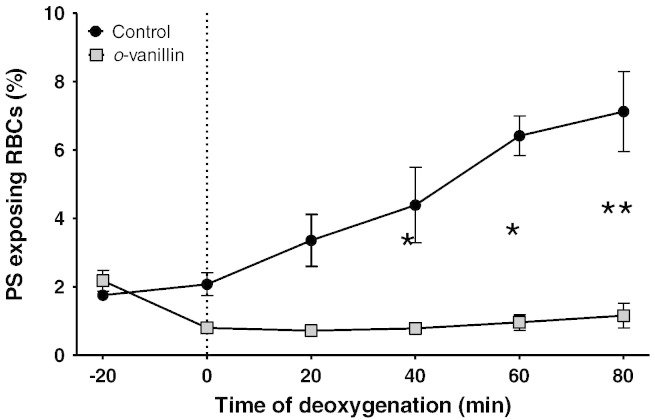
The effect of *o*-vanillin on deoxygenation-induced phosphatidylserine (PS) exposure in RBCs from patients with SCD. Suspensions of RBCs (0.5% Hct) were deoxygenated in the standard saline (1.1 mM [Ca^2 +^]_o_) in Eschweiler tonometers. Control RBCs (black circles) or in the presence of 1.25 mM *o*-vanillin (grey squares) were incubated in air for 20 min prior to the beginning of deoxygenation. Symbols represent mean ± S.E.M. for *n* = 6. * *p* < 0.02, ** *p* < 0.005 (Student's *t* test).

The effects of *o*-vanillin (5 mM) were then tested on the main cation pathways which mediate solute loss and dehydration of RBCs from SCD patients, under fully oxygenated and fully deoxygenated conditions. Results are shown in Fig. 2 for RBCs from homozygous (HbSS) patients. In the presence of *o*-vanillin, KCC in oxygenated RBCs was substantially inhibited (by about 75%). Pre-treatment with *o*-vanillin for 30 min prior to flux measurement produced a slight increase in inhibition. In these RBCs, KCC activity was reduced by about half by deoxygenation and this residual oxygen-insensitive component of KCC was also sensitive to *o*-vanillin (inhibition of this component of KCC activity was 73 ± 13% without pre-treatment, means ± S.E.M., *n* = 5). For P_sickle_ activity, the effect of *o*-vanillin in the absence of pre-treatment was insignificant. Following pre-incubation with *o*-vanillin, however, P_sickle_ activity was inhibited by about 50% (Fig. 2). Consistent with an inhibitory effect on P_sickle_, deoxygenation-induced phosphatidylserine exposure was completely inhibited by incubation in the presence of *o*-vanillin (Fig. 3). Effects on deoxygenation-activated Gardos channel activity were also determined. As for KCC, substantial inhibition (about 80%) was observed without pre-treatment (Fig. 2). In these experiments and similar to findings shown in Fig. 1, following complete deoxygenation sickling was unaffected by the presence of *o*-vanillin (being 98 ± 4%, mean ± S.E.M., *n* = 5, of control values in the absence of *o*-vanillin). It would therefore appear that *o*-vanillin can substantially inhibit both KCC and the Gardos channel without any inhibition of HbS polymerisation and sickling. Similar findings were obtained using RBCs from the second main genotype of SCD patients, heterozygous HbSC individuals, with KCC and Gardos channel activities reduced to < 20% their magnitude in the absence of *o*-vanillin (5 mM).

### The effect of *o*-vanillin on N-ethylmaleimide-treated RBCs from SCD patients

**Fig. 4:**
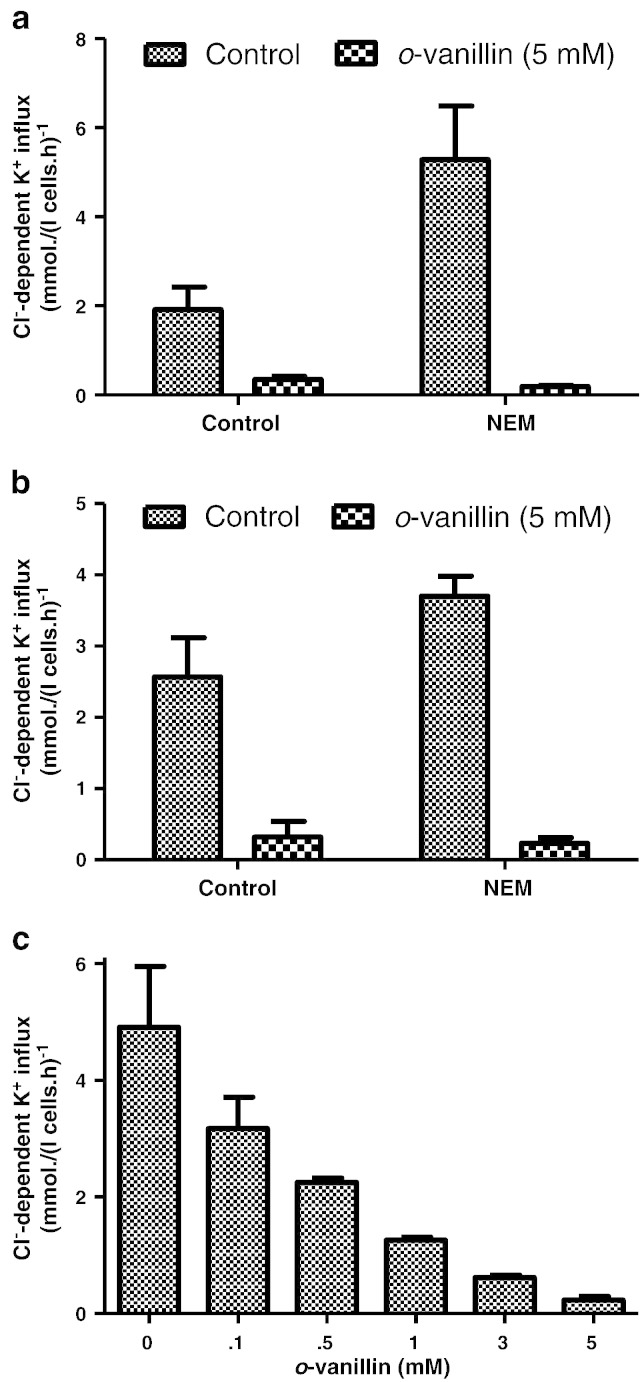
Effect of *o*-vanillin on KCC activity in *N*-ethylmaleimide (NEM)-treated RBCs from patients with SCD. Oxygenated RBCs were pre-treated with NEM (1 mM) for 30 min at 37 °C. Controls were handled similarly but without addition of NEM. Cl^−^-dependent K^+^ influx in mmol (l cells h)^−1^ was then measured in the absence or presence of *o*-vanillin at the concentrations indicated. (a) & (b) KCC activity without or with 5 mM *o*-vanillin in RBCs from HbSS or HbSC individuals, respectively; (c) concentration dependence of inhibition of KCC activity in RBCs from HbSS individuals. Histograms represent means ± S.E.M., n = 3.

KCC activity is controlled by protein phosphorylation, involving cascades of regulatory protein kinases (PK) and phosphatases (PP), on both serine–threonine and tyrosine residues [26,27]. The inhibitory action of *o*-vanillin could therefore be mediated via this cascade. To investigate this possibility, RBCs were pre-treated with *N*-ethylmaleimide (NEM; 1 mM), a thiol-reacting reagent which activates KCC activity and abolishes its sensitivity to (de)phosphorylation [26]. Under these conditions, substantial inhibition of KCC activity by *o*-vanillin (5 mM) was still observed in RBCs from both HbSS and HbSC individuals (Figs. 4a & b). The IC_50_ for *o*-vanillin on KCC activity in NEM-treated RBCs from HbSS patients was about 0.3 mM (Fig. 4c). It would therefore appear that the action of *o*-vanillin on KCC is not via the regulatory phosphorylation cascade but more likely directly on the transporter itself.

### The effect of *o*-vanillin on A23187-treated RBCs from SCD patients

**Fig. 5:**
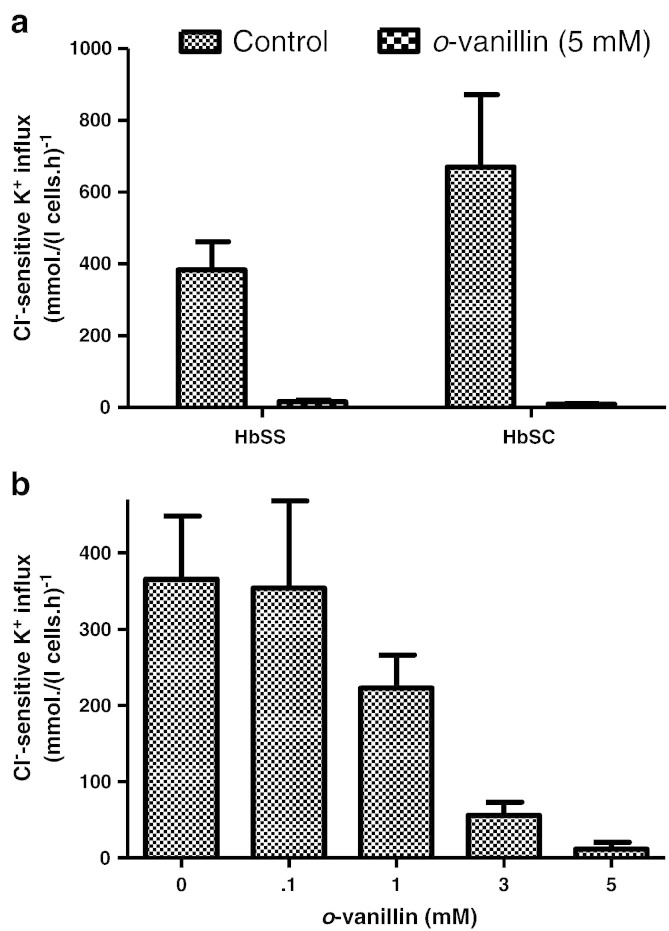
Effect of *o*-vanillin on Gardos channel activity in A23187-treated RBCs from patients with SCD. Oxygenated RBCs were pre-treated with A23187 (10 μM) for 30 min at 37 °C. CLT (5 μM)-sensitive K^+^ influx in mmol (l cells h)^−1^ was then measured in the absence or presence of *o*-vanillin at the concentrations indicated. (a) Gardos channel activity without or with 5 mM *o*-vanillin in RBCs from HbSS or HbSC individuals, as indicated; (b) concentration dependence of inhibition of Gardos channel activity in RBCs from HbSS individuals. Histograms represent means ± S.E.M., *n* = 3.

In the previous experiments (Fig. 2), Gardos channel activity was activated by deoxygenation, following Ca^2 +^ entry through the deoxygenation-induced P_sickle_ activity. Under these conditions, the magnitude of the CLT-sensitive K^+^ influx was modest, at about 6 mmol (l cells h)^ −1^, considerably below the peak values achievable in RBCs following full activation of the channel. Using the ionophore A23187 to load RBCs with Ca^2 +^ [28] can achieve activities of several hundred mmol (l cells h)^ −1^. In fully oxygenated conditions, RBCs were incubated with A23187 (4 μM) and an extracellular Ca^2 +^ of 10 μM to give a free intracellular Ca^2 +^ of about 20 μM, given the usual Donnan ratio of about 1.4 [29]. Gardos channel activity of up to 700 mmol K^+^ (l cells h)^−^ was achieved which was still largely abolished in the presence of 5 mM *o*-vanillin in both HbSS and HbSC RBCs (Fig. 5a). The IC_50_ was about 1 mM whilst inhibition at 5 mM was about 95% (Fig. 5b). Thus *o*-vanillin was also able to inhibit Gardos channel activity directly, and again this was irrespective of any effect on HbS polymerisation or P_sickle_ activity.

### The effect of *o*-vanillin on Na^+^/K^+^ pump activity in RBCs from SCD patients

**Fig. 6:**
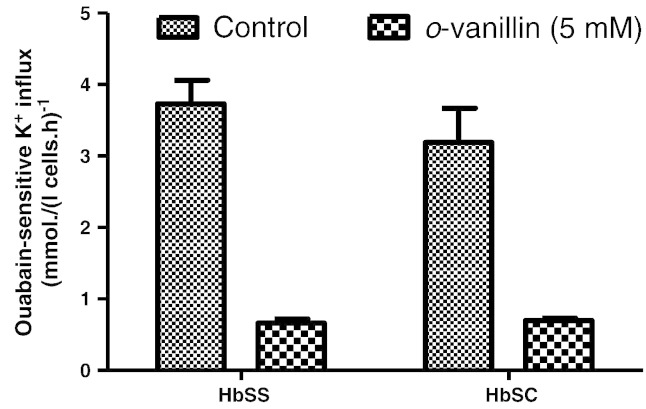
Effect of *o*-vanillin on Na^+^/K^+^ pump activity in RBCs from patients with SCD. Ouabain (100 μM)-sensitive K^+^ influx in mmol (l cells h)^−1^ was measured in fully oxygenated RBCs from HbSS or HbSC individuals, as indicated. Histograms represent means ± S.E.M., *n* = 3.

The Na^+^/K^+^ pump, although of much lower capacity and volume regulatory significance than KCC, P_sickle_ and the Gardos channel, is nevertheless also able to mediate net solute efflux from RBCs including those from SCD patients. Thus, it may also participate in dehydration, particularly following Na^+^ loading via P_sickle_. Previously, it has been shown that phenylalanine benzyl esters, which share some structural similarities with *o*-vanillin, inhibit the Na^+^/K^+^ pump of RBCs. We therefore investigated the effects of *o*-vanillin on this pump. Results are shown in Fig. 6. For RBCs from both HbSS and HbSC patients, control Na^+^/K^+^ pumping activity gave an ouabain-sensitive K^+^ influx of about 4 mmol (l cells h)^−^, around 50% higher than usually observed in normal RBCs. In the presence of *o*-vanillin, inhibition of pump activity in RBCs from both genotypes was about 80% (Fig. 6).

### Effect of *o*-vanillin on RBCs from normal individuals

**Fig. 7:**
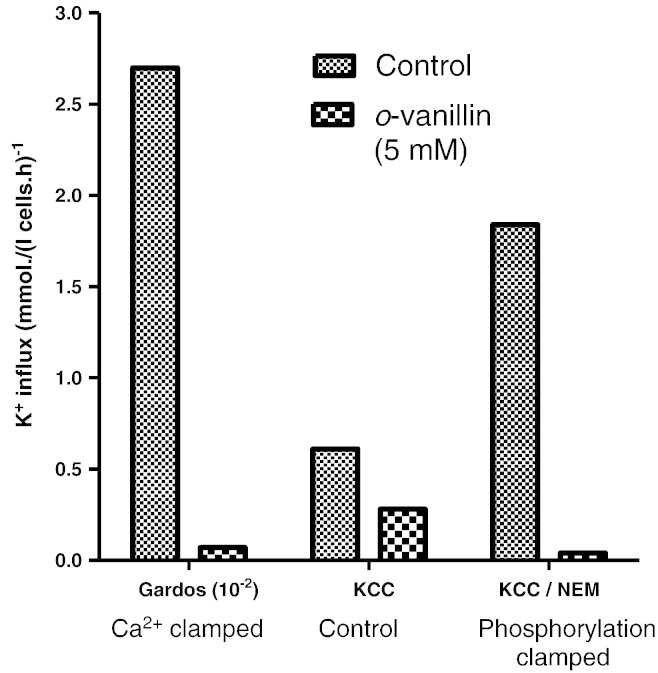
Effect of *o*-vanillin on KCC and Gardos channel activity in RBCs from normal HbAA individuals. Transporter activity was measured as described in legends to Figs. 4 and 5 in control RBCs or RBCs treated with 5 mM *o*-vanillin during the period of flux measurement. CLT (5 μM)-sensitive K^+^ influxes in mmol (l cells h)^−1^ were taken as a measure of Gardos channel activity and Cl^−^-dependent K^+^ influxes as a measure of KCC activity. For Gardos channel, RBCs were treated with A23187 and [Ca^2 +^]_o_ of 10 μM and fluxes presented as ×10^− 2^ of their actual magnitude; for KCC, RBCs were untreated or exposed to 1 mM NEM prior to flux measurement. Histograms represent means ± S.E.M., *n* = 3.

To ascertain further whether HbS was involved in its effects on K^+^ permeability, *o*-vanillin was also tested on KCC and Gardos channel activity on RBCs from normal HbAA individuals (Fig. 7). As in RBCs from SCD patients, KCC was substantially inhibited in HbAA RBCs whether activated by swelling or by NEM (Fig. 7). Similarly, in A23187-treated normal RBCs, there was almost complete inhibition of Gardos channel activity (Fig. 7).

### Effect of *o*-vanillin on cell volume and K^+^ efflux

In the final set of experiments, the effect of *o*-vanillin was tested on RBC volume and K^+^ efflux. Following 60 min deoxygenation, RBCs from HbSS patients fell from 1.88 ± 0.01 to 1.74 ± 0.05 ml/g dcs in the absence of *o*-vanillin, and to 1.52 ± 0.01 in its presence. Notwithstanding its inhibitory effects on the defined K^+^ pathways, *o*-vanillin was therefore found to cause a reduction in RBC volume. K^+^ efflux was also found to increase from 0.091 ± 0.012 h^−1^ to 0.192 ± 0.12 h^−1^.

## Discussion

The present ms presents the first evidence that, as well as reducing HbS polymerisation and sickling, the aromatic aldehyde *o*-vanillin also directly inhibits the main cation pathways which contribute towards dehydration of HbS-containing RBCs. In particular, *o*-vanillin directly inhibited both the KCl cotransproter (KCC) and the Ca^2 +^-activated K^+^ channel (the Gardos channel) of RBCs from both HbSS and HbSC SCD patients and normal individuals, and also the Na^+^/K^+^ pump. Results also indicate partial inhibition of the third main pathway involved in sickle cell dehydration, the deoxygenation-induced cation conductance sometimes termed P_sickle_, independent of an effect on RBC sickling. These findings indicate that aromatic aldehydes may protect sickle cells by two distinct and potentially synergistic mechanisms: by interacting directly with HbS to inhibit polymerisation and also independently of any effect on Hb by reducing cation loss, maintaining RBC hydration and hence reducing the concentration of HbS. The latter effects should be taken into account when designing potential therapeutic members of these compounds.

Despite being the commonest severe inherited disorder affecting millions of people worldwide, treatment for SCD remains problematical. As complications of SCD follow from polymerisation of HbS and RBC sickling, there has been considerable effort directed at discovering novel anti-sickling reagents. Many of these have been designed to interact directly with HbS, to stabilise the oxy conformation (increasing O_2_ affinity) and to inhibit polymerisation [9–11].

Various carbonyl compounds were shown to reduce RBC sickling over forty years ago, with aromatic aldehydes more effective than aliphatic aldehydes [9]. The reactive aldehyde group is thought to form Schiff bases with Hb amino groups, particularly the terminal α1val, and thereby increase O_2_ affinity. Amongst the most potent of the aromatic aldehydes tested was *o*-vanillin [9,30]. Its isomer *p*-vanillin (vanillin) is also thought to react with αHis103 to promote the oxy conformation, with possible other interactions at key sites of polymer contact (βHis116 and βHis117). In vivo, although vanillin itself is poorly absorbed, a pro-drug MX-1520 was shown to protect sickle rats against hypoxia [31].

A number of substituted benzaldehydes, notably 12C79 (also known as BW12C or valerosol) and 589C80 (BWA589C or tucaresol), were also designed to act in a similar manner but with greater binding ability to Hb [32–34]. In experiments involving cyclical deoxygenation and re-oxygenation of sickle cells in vitro both were effective in maintaining intracellular K^+^, high MCV and better deformability [35]. Combination of these benzaldehydes to act via reducing HbS depolymerisation along with direct inhibition of the Gardos channel with clotrimazole and nitrendipine was synergistic in protecting sickle RBCs from shrinkage and K^+^ loss during episodes of cyclical deoxygenation [36]. In clinical trials, 12C79 (valerosol) was effective in increasing O_2_ affinity of Hb both in normal HbAA individuals [37] and SCD patients [38] but had a rather short half life. Although 589C80 (tucaresol) with its longer half life and ability to improve haematological parameters in sickle patients, side-effects included fever and cervical lymphadenopathy [39].

More recently, attention has turned to other potential anti-sickling reagents. Amongst these are the heterocyclic aldehydes (furanic compounds). They too have a similar action binding to α1val and also probably disrupting a key salt bridge with the C-terminal carboxyl group of arg141α [11]. One of them, 5HMF was found to be several times more potent than vanillin in inhibiting sickling [40]. It also protected sickle mice from hypoxia [11]. These findings are very encouraging and currently, 5HMF is the subject of clinical trials in SCD patients.

During the development of these HbS reactive compounds, any effects on RBC permeability have received rather less attention. There are indications, however, that this might be significant. Thus, L-phenylalanine benzyl ester, which was found to reduce sickling, appears to partition into the RBC membrane and non-specifically inhibits transport systems including the Na^+^/K^+^ pump, the cation cotransporters (probably the Na^+^–K^+^–2Cl^−^ cotransporter, NKCC) and the anion exchanger (AE1) whilst also increasing passive cation leaks [12]. No information is available on the aromatic aldehydes.

The current results provide the first evidence that *o*-vanillin directly inhibits the RBC KCC, Gardos channel and P_sickle_. As reported, *o*-vanillin was found to increase O_2_ affinity and inhibit sickling, but their effects on these permeability pathways do not depend on this action. Thus, for KCC and Gardos channel, inhibition also occurred when RBCs were treated with either the sulphydryl reacting reagent NEM or the Ca^2 +^ ionophore A23187, manipulations which bypass any anti-sickling action of *o*-vanillin. The Na^+^/K^+^ pump was also inhibited by *o*-vanillin. Although this raises the possibility that it acts non-specifically, as suggested for the phenylalanine benzyl esters [12], perhaps by partitioning into the membrane and destabilising the transporters, the much reduced effect of its isoform, *para*-vanillin (or usually simply vanillin) argues against this. 5HMF, currently in clinical trials in SCD patients, was different in effect, at least in the transport assays carried out in this work. Nevertheless, present findings indicate that it is possible to design aromatic aldehydes which combine a direct inhibitory effect on HbS polymerisation together with favourable effects on reduction of RBC permeability to thereby increase RBC hydration. These dual effects may potentiate their ability to ameliorate the complications of SCD.

## Conflict of interest

There are no conflicts of interest to declare.

## Contribution

AH carried out most experiments with assistance from UMC and OTG. Study was designed by JSG, DCR and ST. Analysis was carried out by AH, UMC and OTG. Manuscript was prepared by JSG, AH and DCR.
